# Changes in intraocular pressure after pharmacologic pupil dilation

**DOI:** 10.1186/1471-2415-12-53

**Published:** 2012-09-27

**Authors:** Joon Mo Kim, Ki Ho Park, So Young Han, Kwan Soo Kim, Dong Myung Kim, Tae Woo Kim, Joseph Caprioli

**Affiliations:** 1 Department of Ophthalmology, Sungkyunkwan University School of Medicine, Kangbuk Samsung Hospital, Seoul, Korea; 2 Department of Ophthalmology, Seoul National University College of Medicine, Seoul National University Hospital, Seoul, Korea; 3 Department of Ophthalmology, Seoul National University College of Medicine, Seoul National University Bundang Hospital, Seongnam, Korea; 4 Department of Ophthalmology, Jules Stein Eye Institute, University of California Los Angeles, Los Angeles, CA, USA

**Keywords:** Mydriasis, Flare, Anterior chamber angle, IOP variation

## Abstract

**Background:**

Intraocular pressure (IOP) may vary according to the change of ocular conditions. In this study, we want to assess the effect and mechanism of pupil dilation on IOP in normal subjects.

**Methods:**

We prospectively evaluated 32 eyes of 32 patients (age; 61.7 ± 8.2 years) with normal open angles under diurnal IOP. IOP was measured every two hours from 9 AM to 11 PM for one day to establish baseline values and was measured again for one day to assess the differences after dilation. To induce dilation, we administered 2.5% phenylephrine and 1% tropicamide every 5 minutes from 8:30 AM to 8:45 AM and for every two hours from 11 AM to 9 PM to keep the pupil dilated. Diurnal IOP, biometry, Visante OCT, and laser flare photometry were measured before and after dilation.

**Results:**

We observed a significant increase in IOP after dilation, 1.85 ± 2.01 mmHg (*p* = 0.002). IOP elevation remained significant until about four hours after dilation. Thereafter, IOP decreased slowly and eventually reached pre-dilation level (*p >* 0.05). Flare values decreased, and the anterior chamber angle became wider after mydriasis.

**Conclusions:**

Dilation of the pupil significantly and incidentally elevated IOP in normal subjects. Further related studies are warranted to characterize the mechanism of the increased IOP after dilation.

## Background

Like many biological parameters, IOP is a dynamic parameter and varies throughout the course of 24 hours, possibly following circadian rhythms. The mean range of diurnal IOP variation is approximately 2 to 6 mmHg in the normal population and 5 to 18 mmHg in glaucoma patients [1,2]. IOP variation can be affected by many factors such as medication, posture, exercise, blinking, eye movements, and Valsalva manoeuvres [3,4]. As such, clinicians are advised to conduct multiple measurements over 24 hours to assess the IOP profiles of at-risk patients.

Mydriatics are regularly used to dilate pupils in patients presenting to ophthalmology clinics for assessment and follow-up of a wide variety of ophthalmic conditions. An increase in IOP has been observed after dilation with topical application of both parasympatholytic and sympathomimetic mydriatics [5-7]. However, the mechanism of IOP elevation after dilation is not clear.

This prospective study was performed to investigate the effect and mechanism of dilation on IOP in normal subjects.

## Methods

This study adhered to the tenets of the Declaration of Helsinki and was approved by the institutional review board of Kangbuk Samsung Hospital in Seoul, Korea. We examined 32 eyes of 32 patients (17 women and 15 men, age; 61.7 ± 8.2 years) who provided informed consent. All subjects were patients scheduled for a bilateral cataract operation who underwent a full ophthalmic examination including visual acuity, Goldmann applanation tonometry, gonioscopy, slit lamp evaluation, fundus biomicroscopy, auto refractometry (RK-F1, Canon, Japan), and pachymetry (4000AP®, Sonomed®, USA). All eyes presented as normal (except cataract) with an open angle by Goldmann three-mirror gonioscopy. Exclusion criteria included the following: high IOP (>20 mmHg) on the visit before dilation; preoperative ocular medication that could influence IOP level; pre-existing ocular pathology such as glaucoma, uveitis, or high myopia; and previous ocular surgery.

IOP was measured in both eyes of each patient by experienced personnel using a Goldmann applanation tonometer every two hours from 9 AM to 11 PM to establish baseline values. On another day (1 ~ 3 months after the baseline test), we induced mydriasis by administering one drop of Mydrin-P (fixed combination of 2.5% phenylephrine and 1% tropicamide, Santen Pharmaceuticals, Osaka, Japan) in the conjunctival sac every 5 minutes from 8:30 AM to 8:45 AM and every two hours from 11 AM to 9 PM to maintain a dilated state. IOP was measured every two hours from 9 AM to 11 PM.

The following variables were assessed before and after dilation: diurnal IOP, anterior segment examination, axial length (AL), anterior chamber depth (ACD), central corneal thickness (CCT), anterior chamber flare with laser flare photometer (FM-500, Kowa, Tokyo, Japan), and anterior chamber angle with Visante OCT (Carl Zeiss Meditec, Dubin, CA, USA). AL and ACD were measured with an IOL Master (Carl Zeiss Meditec, Dubin, CA, USA). CCT was measured with a hand-held ultrasonic pachymeter, and the average of three readings was recorded.

The Visante OCT was used to perform anterior chamber angle width measurements every two hours before mydriasis and after instillation of mydriatics from 9 AM to 9 PM. The average of three consecutive readings of the mean angle value at 3 and 9 o’clock was included in analysis. Laser flare photometry was performed once before mydriasis and every two hours after instillation of mydriatics, from 9 AM to 5 PM. The laser flare photometer quantifies anterior chamber protein (flare) and particles (cells) by measuring light scattering of a helium-neon laser beam projected into the anterior chamber [8]. A single experienced investigator examined each subject five times in series and recorded the mean value of the five measurements. All examinations were performed in a hospital setting. To reduce bias, a different measuring technician, data collector, and statistical analyst participated in a masked fashion. The data were analyzed using PASW statistics 17.0 (SPSS, Inc., Chicago, IL, USA), and differences in values were assessed by paired t-test. A *p*-value of less than 0.05 was considered statistically significant.

## Results

The mean pre-dilation IOP was 11.48 ± 2.85 mmHg. The mean post-dilation IOP was 12.36 ± 2.58 mmHg. This change was statistically significant (*p* = 0.005). The maximum IOP also significantly increased from a mean pre-dilation level of 13.10 mmHg ± 2.91 to a post-dilation level of 14.96 ± 3.25 mmHg (*p* < 0.001). However, there was no significant difference between the minimum IOP before (10.50 ± 2.74 mmHg) and after (10.50 ± 2.35 mmHg) dilation (*p =* 0.978).

Regarding diurnal IOP variation, the mean pre-dilation value was 2.60 ± 1.14 mmHg, and the mean post-dilation value was 4.45 ± 2.01 mmHg. The difference in the mean change of diurnal IOP variation was 1.85 ± 2.01 mmHg, and this change was statistically significant (*p* = 0.002). Diurnal IOP was elevated in 22 eyes (68.9%), decreased in two, and unchanged in eight. We noted a non-significant IOP increase at 9 AM, 30 minutes after dilation. IOP was significantly increased at 11 AM and 1 PM (Table 1). Maximum IOP levels were reached at 11 AM, and after 3 PM, the IOP did not significantly differ (*p* > 0.05) from pre-dilation levels.

**Table 1:** **The variation of intraocularpressure (IOP) before andafter pupil dilation**

**Time**	**IOP before dilation(mmHg)**	**IOP after dilation(mmHg)**	**p-value**
9	12.35 ± 3.06	12.54 ± 2.82	0.898
11	12.25 ± 3.12	14.33 ± 3.65	0.001*
13	11.49 ± 2.68	13.42 ± 3.30	<0.001*
16	11.57 ± 2.95	12.29 ± 2.58	0.051
17	11.70 ± 2.83	11.85 ± 2.30	0.604
19	11.42 ± 2.96	11.80 ± 2.82	0.372
21	11.39 ± 2.97	11.39 ± 2.46	0.799
23	11.32 ± 2.76	11.32 ± 2.58	0.944

* : p-value < 0.05.

**Figure 1:**
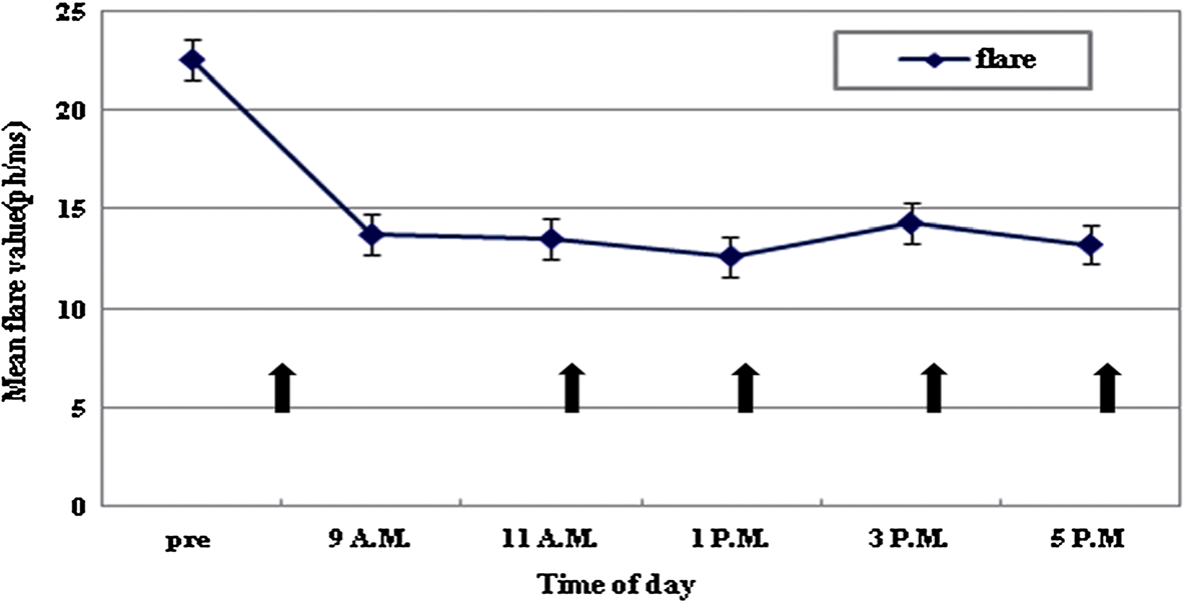
**Laser flare photometry values.** Flare values decreased significantly after dilation and remained low. The arrows indicate the time mydriatics were given. Intraocular pressure (IOP) at 9A.M. was measured before the mydriatics were instilled. To maintain pupil dilation, mydriatics were instilled every 2 hours until 9 PM, just after IOP measurement.

**Figure 2:**
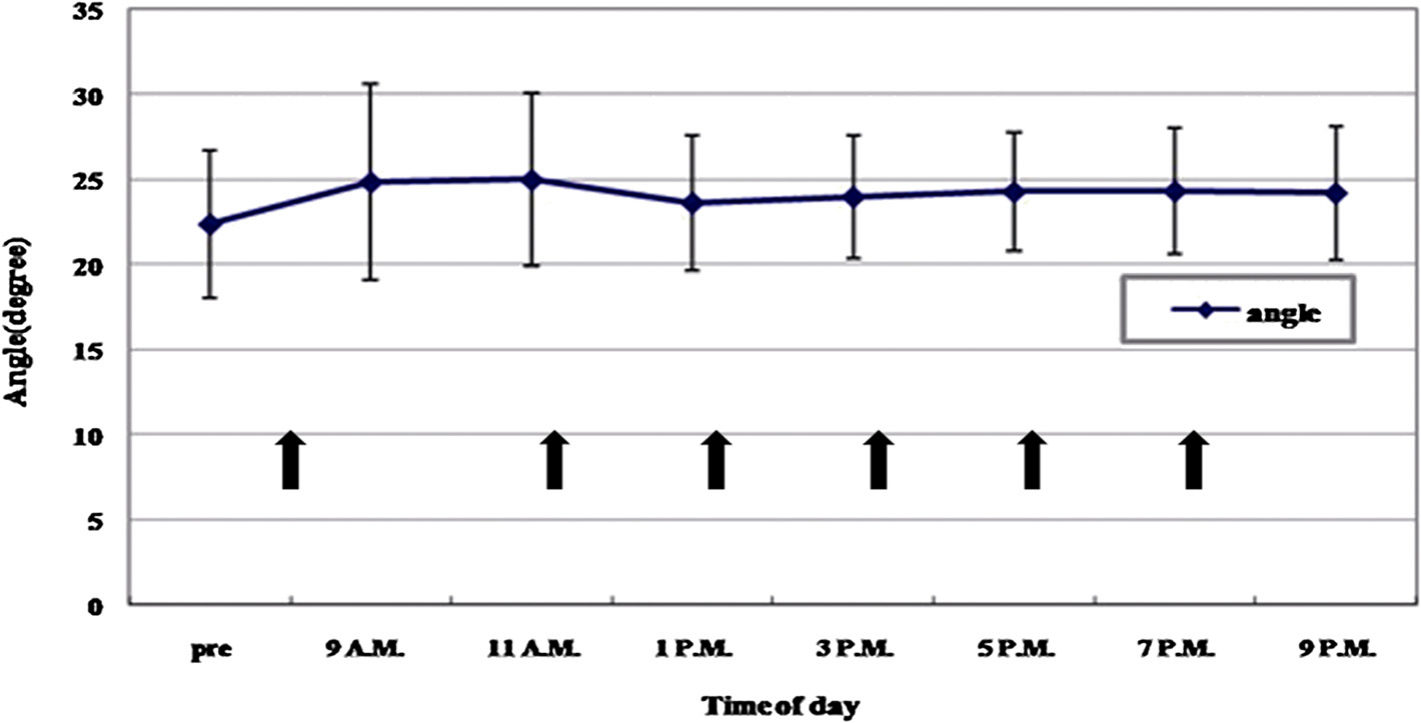
**Anterior chamber angle widthsmeasured by Visante OCT**. Anterior chamber angles increased significantly after dilation.

The flare value decreased after dilation and remained constant (Figure 1). The width of the anterior chamber angle increased significantly after dilation, and this state was maintained while the pupil was dilated (*p* < 0.001) (Figure 2). After dilation, we noted significantly increased ACD values (3.20 ± 0.45 mm), as compared with initial values (3.09 ± 0.48 mm) (*p* < 0.001). Mean pupil diameter increased from 2.975 ± 0.498 mm to 6.725 ± 0.717 mm 2 hours after dilation and 6.793 ± 0.616 mm after 8 hours, but these changes were not statistically significant (p > 0.05). There was no significant variation of pupil size in either time interval (p > 0.05).

IOP measurements after dilation were not related to the mean pre-dilation IOP, AL, or diurnal IOP value according to the results of multivariate analysis (range of *p*-values: 0.232-0.966). Changes in the mean CCT and axial length measurements before and after dilation were not statistically significant.

No patients developed clinically significant (>10 mmHg) sustained increases in IOP. Only one patient experienced a rise in IOP to a level greater than 21 mmHg after dilation. The patient’s pressure dropped after an additional two hours with no medical intervention. This patient exhibited the highest pre-dilation IOP level of all of our patients but did not have any other distinctive findings.

## Discussion

Tropicamide is an anticholinergic drug, and phenylephrine is an alpha-1-adrenergic agent. These agents are commonly used together to achieve mydriasis for fundus examination [9-11]. It has previously been recognized that pharmacologic mydriasis can cause an elevation in IOP. According to one study, 1-2% of healthy persons display a pressure elevation of 6 mmHg or more after treatment with 1% cyclopentolate [5]. Harris and Galin showed that 33% of miotic-treated patients with open angle glaucoma respond to cycloplegics with a pressure elevation [12]. Blake et al. found that significant pressure elevation occurred in 32% of open angle glaucoma patients following dilation with 2.5% phenylephrine and 1% tropicamide [13].

The mechanism responsible for IOP elevation after dilation is unclear. Mydriatic agents can cause increases in IOP that may be related to decreasing aqueous outflow resulting from decreased traction on the trabecular meshwork due to ciliary muscle paralysis [14,15]. Under the previously mentioned conditions, we can presume, if the dilated state is maintained, increased IOP may be preserved. The results of our study suggest a different explanation. IOP was found to be significantly increased at four hours and six hours after pupil dilation during preserved pharmacologic mydriasis and slowly decreased after that time. After dilation, the ACD deepened, the anterior chamber angles widened, and the anterior chamber flare decreased. Harris showed that a narrow angle was a crucial factor that predisposed patients to acute IOP elevation, but IOP elevation has been found to occur in eyes that do not have narrow angles [16]. Valle reported that the key characteristic separating responders to cyclopentolate from non-responders was a difference in the inflow rate, [17] whereas the outflow rate through the trabecular meshwork decreased with cyclopentolate in all patients studied. The only statistically significant difference between the two groups was that the inflow decreased in non-responders but increased slightly in responders. In our study, the mean IOP values decreased in two patients after dilation. Temporary imbalance of aqueous flow may have an effect on these patients. Also, dilation can cause a greater anterior chamber depth and a wider contact region between the trabecular meshwork and the aqueous. There may be small amount of flare before the dilation, and a small amount of flare may also occur due to rubbing between iris and lens when the pupil begins dilation. Thus, IOP may not increase after dilation. Further evaluation with a larger population based study may be needed.

Iris pigment liberation into the anterior chamber and subsequent obstruction of the trabecular meshwork has also been noted as a possible mechanism responsible for the increase in IOP [18-20]. Kristensen showed that 48% of eyes with open angle glaucoma showed a rise in pressure of 8 mmHg or more after dilation, and all elevations were associated with marked pigment elevation [19]. Valle demonstrated IOP elevations of up to 20 mmHg after dilation with 1% cyclopentolate, all of which were accompanied by pigment liberation [20].

In our study, we investigated aqueous flare and anterior chamber angles before and after mydriasis to help determine the aetiology of increased IOP. We demonstrated that anterior chamber angles widened with dilation, which is possibly due to the posterior pull of the dilated iris-lens diaphragm, leading to a deep anterior chamber. The reduction in IOP after 3 pm may be explained by the widened anterior chamber angle and improvement of the aqueous outflow facility. A decreased resistance to aqueous outflow may be expected from deepening of the anterior chamber, which creates a larger surface area between the trabecular meshwork and the aqueous humour [21]. However, IOP increased just after dilation, and we assessed the variation in flare after dilation. Flare values decreased after mydriasis (*p* < 0.01). The flare value decreased just after dilation and remained decreased while the pupil was dilated. It is possible that, just after mydriasis, the flare may be increased by iris pigment liberation or by protein, but crowding in the angle and subsequent interruption through the outflow facility of the trabecular meshwork can explain the elevation of intraocular pressure. Jewelewicz et al. reported similar results in pigment dispersion syndrome cases [22]. The maximal pigment liberation was reached 30 to 60 minutes after mydriasis, but peak IOP was reached about 90 minutes after mydriasis, when the anterior chamber pigment was decreasing. Our cases have some differences. The flare decreased 30 minutes after instillation of mydriatics. This might be because the subjects of our study were normal, and normal subjects may have a different response to dilation than pigment dispersion syndrome patients. Another possibility was that we used a combination drug. The included phenylephrine could increase the clearance of flare/pigment. Racial differences (only Koreans were included in our study) should also be considered. Also, we took measurements of fully dilated eyes, which can explain why we did not observe more iris pigment liberation due to lack of contact between the iris and lens. The mydriasis effect may have increased vessel stability and decreased flare.

Laser flare photometry is an objective, quantitative method that enables accurate measurement with very high sensitivity and reproducibility. Guillen-Monterrubio et al. reported no significant differences in flare values measured by flare photometer between right and left eyes or between men and women [23].

There are some limitations in this study. Ocular parameters, such as corneal thickness and shape, [24,25] anterior chamber depth, [26] and axial length [25] are known to undergo significant diurnal changes. We did not control these factors. Angle and ACD are affected by many external influences such as near vision or distance vision, so it is difficult to measure the diurnal effect. Also, corneal thickness may be changed due to epithelial oedema by the repeated application of eye drops causing the possible under measurement of IOP. This can affect the decreased IOP of late diurnal measurements, but it is difficult to calculate the cushion effect caused by epithelial oedema. Further study is needed. IOP may change as either diurnal variation or seasonal change. Qureshi IA et al. reported that IOP tends to increase in the winter [27]. The longest time interval of IOP measurement among the patients was three months, which could possibly affect the variability.

## Conclusions

According to the results of this study, pupil dilation caused an elevation of IOP. The elevation of IOP was significant until four to six hours after dilation. Afterwards, IOP decreased slowly until it reached pre-dilation level. Further related studies in glaucoma patients are warranted to characterize the mechanism of increased IOP after dilation in a diseased state.

## Competing interests

JMK; none, KHP; none, SYH; none, KSK; none, TWK; none, DMK; none, JC has received consultant fees and honoraria from Allergan.

## Authors' contributions

Literature screening and selection was performed by JMK and KSK. JMK, KHP, TWK, DMK and JC participated in the design of the study. Data collection was done by SYH and KSK, and SYH and KSK performed the statistical analysis. Preparation of the first draft of the manuscript was done by JMK. Critical revision was performed by KHP, SYH, KSK, TWK, DMK, and JC, and approval of the final version of the manuscript was performed by JMK, KHP, SYH, KSK, TWK, DMK, and JC.

## Pre-publication history

The pre-publication history for this paper can be accessed here:

http://www.biomedcentral.com/1471-2415/12/53/prepub
